# Downregulation of inhibitory SRC Homology 2 Domain-containing Phosphatase-1 (SHP-1) leads to recovery of T cell responses in elderly

**DOI:** 10.1186/1478-811X-12-2

**Published:** 2014-01-09

**Authors:** Aurélie Le Page, Carl Fortin, Hugo Garneau, Nancy Allard, Krassimira Tsvetkova, Crystal Tze Ying Tan, Anis Larbi, Gilles Dupuis, Tamas Fülöp

**Affiliations:** 1 Research Center on Aging, Faculty of Medicine and Health Sciences, Université de Sherbrooke, 1036 rue Belvedere sud, Sherbrooke, J1H 4C4, Quebec, Canada; 2 Division of Pneumology, Department of Medicine, Université de Sherbrooke, 3001, 12e Avenue Nord, Sherbrooke, J1H 5N4, Quebec, Canada; 3 Division of Geriatrics and Research Center on Aging, Faculty of Medicine and Health Sciences, Université de Sherbrooke, 375 Argyll Street, Sherbrooke, J1J 3H5, Quebec, Canada; 4 Singapore Immunology Network (SIgN), Agency for Science Technology and Research (A*STAR), 8A Biomedical Grove, Singapore, 138648, Singapore; 5 Department of Biochemistry and Graduate Program in Immunology, Faculty of Medicine and Health Sciences, Université de Sherbrooke, 3001, 12e Avenue Nord, Sherbrooke, J1H 5N4, Quebec, Canada

**Keywords:** Aging, Confocal microscopy, Lck, Lipid rafts, Protein tyrosine kinases, Protein tyrosine phosphatases, SHP-1, Lck, T cells, TCR/CD28 signaling

## Abstract

**Background:**

Immune responses are generally impaired in aged mammals. T cells have been extensively studied in this context due to the initial discovery of their reduced proliferative capacity with aging. The decreased responses involve altered signaling events associated with the early steps of T cell activation. The underlying causes of these changes are not fully understood but point to alterations in assembly of the machinery for T cell activation. Here, we have tested the hypothesis that the T cell pool in elderly subjects displayed reduced functional capacities due to altered negative feedback mechanisms that participate in the regulation of the early steps of T cell activation. Such conditions tip the immune balance in favor of altered T cell activation and a related decreased response in aging.

**Results:**

We present evidence that the tyrosine phosphatase SHP-1, a key regulator of T cell signal transduction machinery is, at least in part, responsible for the impaired T cell activation in aging. We used tyrosine-specific mAbs and Western blot analysis to show that a deregulation of the Csk/PAG loop in activated T cells from elderly individuals favored the inactive form of tyrosine-phosphorylated Lck (Y505). Confocal microscopy analysis revealed that the dynamic movements of these regulatory proteins in lipid raft microdomains was altered in T cells of aged individuals. Enzymic assays showed that SHP-1 activity was upregulated in T cells of aged donors, in contrast to young subjects. Pharmacological inhibition of SHP-1 resulted in recovery of TCR/CD28-dependent lymphocyte proliferation and IL-2 production of aged individuals to levels approaching those of young donors. Significant differences in the active (Y394) and inactive (Y505) phosphorylation sites of Lck in response to T cell activation were observed in elderly donors as compared to young subjects, independently of CD45 isoform expression.

**Conclusions:**

Our data suggest that the role of SHP-1 in T cell activation extends to its increased effect in negative feedback in aging. Modulation of SHP-1 activity could be a target to restore altered T cell functions in aging. These observations could have far reaching consequences for improvement of immunosenescence and its clinical consequences such as infections, altered response to vaccination.

## Background

Aging is generally associated with an impairment of the immune response, a phenomenon globally referred to as immunosenescence [1-4]. For instance, there is reduced production of IL-2 and impaired proliferation of T lymphocytes of elderly individuals [5], as well as a series of altered signaling events associated with the early steps of T cell activation reviewed in [6,7]. Immunosenescence is observed even in healthy aged individuals [8] and has been shown to affect the innate and adaptive arms of the immune response [9,10]. Although the mechanism of immunosenescence remains not fully understood, it has been clearly established that T cells of aged individuals display reduced clonal expansion that translates into increased susceptibility to infectious diseases [11], impaired responses to vaccination, [12,13], increased susceptibility to cancer [6,7,14,15] and autoimmune diseases [2,16-18] and, chronic inflammatory diseases such as Alzheimer’s disease and atherosclerosis [19,20].

T cell activation involves initial recognition of antigenic epitopes presented by professional APC within the context of MHC class I or class II molecules. Close contact between T cells and APC results in assembly of a supramolecular structure called the immune synapse (IS) [21-23]. IS formation is modulated through dynamic movements of lipid rafts [24-26] that facilitate local clustering of the essential components of T cell signaling [27]. Interactions between TCR and epitope-loaded MHC I or II and with CD8 or CD4 respectively, provide the first signal that leads to a cascade of events resulting from the assembly of the signal transduction machinery or signalosome [28]. However, a second signal has to be provided by occupation of co-stimulation molecules of activation [29-33]. Combination of TCR and CD28 signaling results in full T cell activation characterized by gene expression, cytokine production, cell proliferation, clonal expansion and, generation of effector and memory functions [28].

From a biochemical standpoint, one of the first events that follow engagement of the TCR by epitope-loaded MHC is the CD45-dependent removal of the phosphate group on tyrosine residue 505 (Y505) of the p56^Lck^ (Lck) Src kinase [34]. Dephosphorylation relieves its cAMP-regulated [35] Csk-dependent inhibition and allows its auto/trans-activation [36]. Activated Lck then targets the TCR-associated ζ homodimer that provide sites for recruitment and activation of the SH2 domain-containing ZAP-70. Activated ZAP-70 tyrosine-phosphorylates several constituents of the signalosome, including scaffold and adaptor proteins, protein and lipid kinases which are essential for Ca^2+^ mobilization [37] and activation of the p21^Ras^ (Ras)/MAP kinase and NF-AT pathways [28,38,39]. On the other hand, engagement of CD28 triggers activation of the NF-κB pathway [40,41]. These activated pathways converge to allow nuclear translocation of the transcription factors AP1, NF-AT and NF-κB and, initiation of expression of a number of genes, including IL-2 and its receptor which are essential for T cell proliferation [42]. Whereas this series of events works in a forward direction to trigger T cell activation and protection against antigenic aggression, complex mechanisms of T cell regulation of activation also operate to prevent uncontrolled responses and autoimmunity and/or immune catastrophe [43,44]. A number of early cytoplasmic components including protein kinases and phosphatases and, adaptor proteins also act as negative feedback regulators of the TCR signalosome [28,44]. A key target for the negative regulation of T cell signaling is Lck. Lck activity is finely tuned by a complex of proteins comprising plasma membrane-embedded protein tyrosine phosphatase CD45 and cytoplasmic protein tyrosine kinase Csk bound to scaffold protein PAG (CBP), and to adaptor protein TSAD [45,46]. In addition, cytoplasmic phosphatases SHP-1 and Lyp (PTPN22) are thought to play a role in the regulation of Lck activity [28,47-49]. Many of the components of the signaling machinery of T cell activation are targets of SHP-1. For instance, SHP-1 removes key phosphate groups on tyrosine residues of Lck [48] and ZAP-70 and that results in loss of activity of these essential protein kinases [50,51]. Furthermore, this regulatory loop of Lck can discriminate between self and non-self as weakly binding ligands predominantly trigger a negative feedback loop leading to rapid recruitment of the tyrosine phosphatase SHP-1, followed by receptor desensitization through inactivation of Lck while, strongly binding ligands efficiently activate a positive feedback circuit involving Lck modification by ERK, preventing SHP-1 recruitment and allowing the long-lasting signaling necessary for gene activation [52,53].

Beside decreased IL-2 production and T cell proliferation, we and others have shown that the composition and function of lipid rafts were altered with aging [54] and that the activation of Lck, Fyn, ZAP70 and LAT was impaired [55,56]. Overall, these observations were consistent with the interpretation that T cell activation and early events in T cell signaling were altered in aged individuals [7]. However, our data did not exclude the possibility that negative regulation of T cell activation through actions of protein tyrosine phosphatases or other mechanisms were also altered. Recently, it has been shown that by modulating later signaling events by phosphatases especially by the DUSP6 repression using miR-181a or specific siRNA and DUSP6 inhibition improved CD4 T cell responses, as evidenced by increased expression of activation markers, improved proliferation and supported preferential T helper type 1 cell differentiation [57,58]. Here, we report that a deregulation of the Csk/PAG/CD45 loop in T cells of elderly subjects favors the maintenance of Lck inhibition through phosphorylation of Y505. We also observed an upregulation of SHP-1 activity and alterations in the dynamic movements of signaling proteins in lipid rafts. Of significance, pharmacological inhibition of SHP-1 resulted in recovery of TCR/CD28-dependent lymphocyte proliferation and IL-2 production in PBMCs of aged individuals. Our data suggest that the regulatory role of SHP-1 in the forward direction of T cell activation through TCR and CD28 extends to its involvement as negative feedback regulator of Lck activity in aging.

## Results

### Differential levels of pLck (Y505) in T cells of young and aged subjects

**Figure 1:**
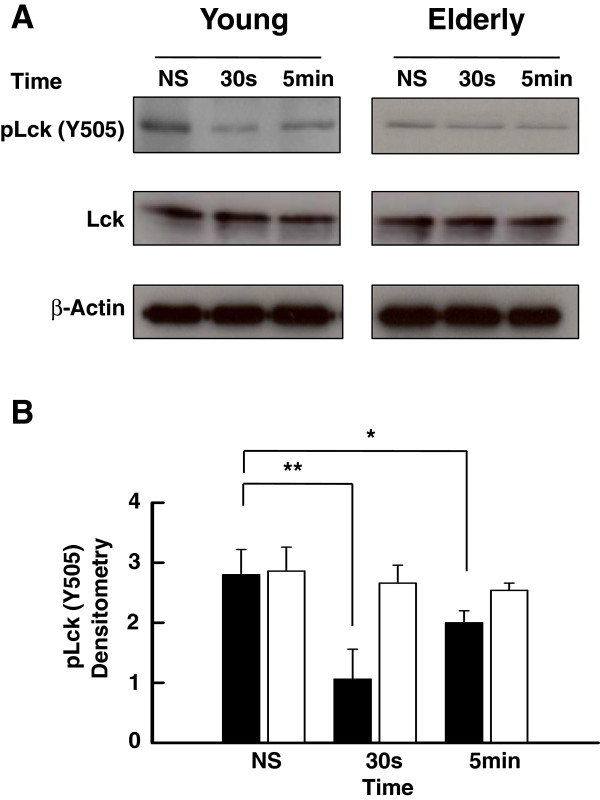
**Western blot analysis of the levels of tyrosine-phosphorylated (Y505) Lck. (A)** Purified T cells from young and elderly donors were left non-stimulated (NS) or exposed to a mixture of anti-CD3 (5 μg/ml) and anti-CD28 (5 μg/ml) mAbs for 30 s or 5 min, as indicated. Cell lysates were sized by SDS-PAGE under reducing conditions, electrotransfered to nitrocellulose membranes and proteins revealed using appropriate mAbs and the chemiluminescence technique. β-Actin was used as control of gel loading. The protein transferred to nitrocellulose membranes were stained with Ponceau to verify that similar amounts of protein had been loaded in each lane. **(B)** Time-related Y505-phosphorylated Lck analyzed by semi-quantitative densitometry and reported in arbitrary units in the case of stimulated (anti-CD3/anti-CD28, 5 μg/ml each) T cells of young (filled columns) and elderly (empty columns). Data are represented as the mean ± SD. Asterisks correspond to statistical significance (Student’s *t*-test) for *p* < 0.05 (*) and *p* < 0.01 (**). Data are representative of one of 20 independent experiments.

We have reported that T cell responses of elderly subjects are impaired at the early proximal steps of T cell activation [6,7], including Lck phosphorylation on Y394 [55]. We hypothesized that the data could be explained by a negative feedback through protein kinases and phosphatases that could play a suppressive role in aging [7]. Whereas a fraction of Lck is in a primed/activated state in T cells [59,60], a major fraction of the kinase is maintained in an inactive state as a result of Csk-dependent phosphorylation of a single tyrosine residue (Y505) located in the C-terminal region. This modification allows the kinase to fold into a closed, inactive conformation [60]. The balance between the different activation states and distribution in plasma membrane microdomains of Lck allows efficient TCR signal transduction in T cells [49,59,60]. Here, we have measured the time-dependent levels of pLck-Y505 in purified T lymphocytes of young and elderly subjects activated through CD3-CD28. Western blots (Figure 1A) showed that the levels of pLck-Y505 rapidly decreased (30 s) following activation of T cells of young individuals (*p* < 0.01) and still remained significantly lower compared to initial levels 5 min after activation (p < 0.05) Figure 1A and B). In contrast, the levels of inactive pLck-Y505 in T cells of elderly subjects slightly, but not significantly decreased over the course of the experiment (Figure 1A and B) suggesting a lack of modulation of pLck-Y505 during T cell activation. Protein levels of Lck are not different between young and elderly subjects and did not change during the course of the experiments. Similar results were obtained by Flow Cytometry (data not shown). Early changes (30 s) observed in young individuals were absent in the case of T cells from elderly. Further gating on CD45RA versus CD45RO T cells of young and elderly subjects did not reveal significant differences for pLck status inside of the age-groups (data not shown). The bulk of these data suggested a differential regulation of Lck in T lymphocytes of young and elderly subjects that depends on the T cell population but not on the CD45RA or CD45RO subpopulations.

### Involvement of PAG phosphorylation in age-related T cell signaling dysfunction

**Figure 2:**
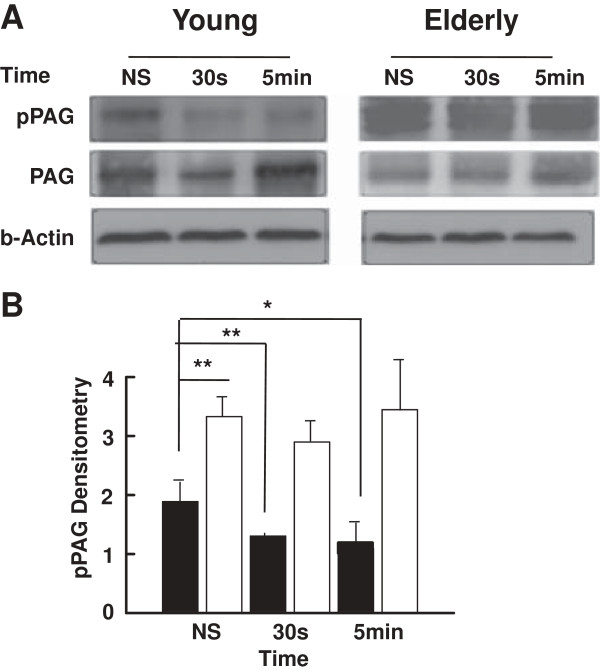
**Western blot analysis of the levels of tyrosine-phosphorylated PAG. (A)** Purified T cells from young and elderly donors were left non-stimulated (NS) or exposed to a mixture of anti-CD3 and anti-CD28 (5 μg/ml each) mAbs for 30 s or 5 min, as indicated. Cell lysates were sized by SDS-PAGE under reducing conditions, electrotransfered to nitrocellulose membranes and proteins revealed using appropriate mAbs and the chemiluminescence technique. β-Actin was used as control of gel loading. The protein transferred to nitrocellulose membranes were stained with Ponceau to verify that similar amounts of protein had been loaded in each lane. **(B)** Protein bands were analyzed by semi-quantitative densitometry and are reported in arbitrary units. Results of T cells of young (filled columns) and elderly (empty columns) donors are shown. Data are represented as the mean ± SD. Asterisks correspond to statistical significance (Student’s *t*-test) for *p* < 0.05 (*), *p* < 0.01 (**). Data are representative of one of 20 independent experiments.

In T cells, phosphorylation of PAG creates docking sites for the SH2 domain-containing protein tyrosine kinase Csk and that enables its recruitment to the plasma membrane. Csk then negatively regulates Src family members Lck and Fyn activity through tyrosine phosphorylation at their C-terminus [46]. Conversely, stimulation of T lymphocytes induces CD45 and other phosphatase-dependent dephosphorylation of pPAG [61], followed by the release of Csk from its proximity with Lck, thus facilitating activation of Lck [62]. In consequence, we have measured the time-dependent levels of pPAG in stimulated lymphocytes of young and elderly subjects. Results (Western blots) revealed a sustained slow decrease of PAG phosphorylation from resting levels in T cells of young subjects following activation (*p* < 0.01 at 30 s and *p* < 0.05 at 5 min) (Figure 2A and B). In contrast, the levels of pPAG in T cells of elderly donors were higher than those of young donors in the resting state (*p* < 0.01) and remained elevated following ligation of CD3-CD28 (*p* < 0.01) (Figure 2A and B). These observations brought additional support to the interpretation that Lck was differentially regulated in T lymphocytes of young and elderly subjects, likely through differential modulation of the levels of pPAG.

### Lipid raft distribution of pPAG and Csk in T cells of young and aged donors

**Figure 3:**
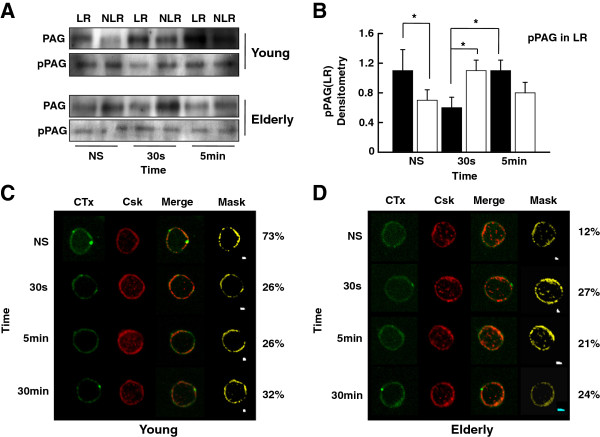
**Western blot and confocal analysis of Csk and tyrosine-phosphorylated PAG in plasma membrane lipid rafts (LR). (A)** Purified T cells were left non-stimulated (NS) or exposed to a mixture of anti-CD3 and anti-CD28 (5 μg/ml each) mAbs for various periods of time, as indicated. Cell lysates were separated on sucrose density gradients and fractions corresponding to lipid rafts (LR) and non-lipid rafts (NLR) were isolated, sized by SDS-PAGE under reducing conditions, electrotransfered to nitrocellulose membranes and proteins revealed using appropriate mAbs and the chemiluminescence technique. β-Actin was used as control of gel loading. The protein transferred to nitrocellulose membranes were stained with Ponceau to verify that similar amounts of protein had been loaded in each lane. **(B)** Protein bands were analyzed by semi-quantitative densitometry and are reported in arbitrary units. Results of T cells of young (filled columns) and elderly (empty columns) donors are shown. Data are represented as the mean ± SD. Asterisks indicate statistical significance (Student’s *t*-test) for *p* < 0.05. **(C and D)** Confocal analysis of the distribution in lipid rafts of cholera toxin B subunit (CTx) and Csk of resting (NS) and T cells activated (TCR-CD28) for various periods of time, as indicated. An illustrative example of young **(C)** and elderly **(D)** individual, merged images and masking are shown. Data are representative of one of a minimum of 12 independent experiments.

We have reported that lipid raft functions are altered with aging [56,63]. Here, we sought to determine whether the distribution of pPAG in lipid rafts was affected in elderly donors. Results showed that pPAG was almost equally located in the lipid raft (LR) and non-lipid raft (NLR) fractions of resting T cells of young subjects (Figure 3A and B). Its distribution to LR significantly decreased 30 s following T cell activation (*p* < 0.05) without any change in the NLR fraction but returned to resting levels 5 min later (Figure 3A and B). The levels of pPAG in the lipid raft fraction of T cells from aged donors were lower than in young subjects in the resting state (*p* < 0.05) (Figure 3A and B). Surprisingly, ligation of CD3-CD28 did not trigger the expected pPAG downregulation in lipid rafts (Figure 3A and B) and even an increased pPAG localization is observed mainly at 30 s (p < 0.05). This is in sharp contrast to what was observed in T cells of young subjects.

Csk has been reported to transiently migrate out of lipid rafts following TCR engagement [61] concomitantly with pPAG migration out of lipid rafts. Confocal microscopy analysis showed that approximately 70% of Csk co-localized in lipid rafts of resting T cells of young donors (Figures 3C). Its distribution in lipid rafts decreased significantly (*p* < 0.006) 30 s after ligation of CD3-CD28 and it remained out of lipid rafts over the course of the experiments, without significant differences (Figures 3C). In marked contrast in the case of aged donors, Csk distribution in resting T cells is less abundant inside LR (Figure 3D). Activation through CD3-CD28 did not result in Csk migration in/out of lipid rafts and its distribution remained nearly unchanged over the course of the experiments (Figures 3D).

### CD45, CD45RA And CD45RO expression, activity in T cells with aging

**Figure 4:**
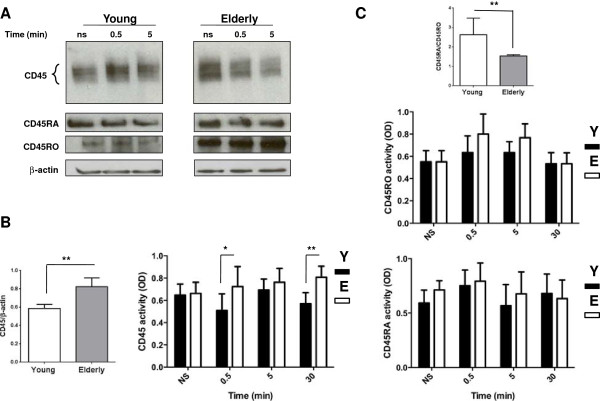
**Western blot analysis and measurement of CD45 and CD45RA and CD45RO activities. (A)** Purified T cells from young and elderly donors were left non-stimulated (NS) or exposed to a mixture of anti-CD3 and anti-CD28 (5 μg/ml each) mAbs for various periods of time, as indicated. Cell lysates were sized by SDS-PAGE under reducing conditions, electrotransfered to nitrocellulose membranes and proteins revealed using an anti-CD45 mAb and the chemiluminescence technique. β-Actin was used as control of gel loading. The protein transferred to nitrocellulose membranes were stained with Ponceau to verify that similar amounts of protein had been loaded in each lane. **(B)** Results of T cells of young (empty columns) and elderly (filled columns) donors are shown. Protein bands were analyzed by semi-quantitative densitometry and are reported in arbitrary units. Left panel shows the CD45/β-actin expression (**, p < 0.01), and right panel shows CD45 activity, in optical density units, of T cell lysate immunoprecipitates determined using 4-nitrophenyl phosphate as a substrate. Results of T cells of young (filled columns) and elderly (empty columns) donors are shown. **(C)** The CD45RA/CD45RO ratio in T cells from young and elderly is shown (top panel). CD45RO activity (middle panel) and CD45RA activity (bottom panel) shown in optical density units in T cell lysate immunoprecipitates determined using 4-nitrophenyl phosphate as a substrate. Results of T cells of young (filled columns) and elderly (empty columns) donors are shown. The protein transferred to nitrocellulose membranes were stained with Ponceau to further verify that similar amounts of protein had been measured for each point of activity. Data are represented as the mean ± SD. Asterisks correspond to statistical significance (Student’s *t*-test) for *p* < 0.05 (*) and *p* < 0.01 (**). Data are representative of one of 20 independent experiments.

CD45 is a type I transmembrane protein tyrosine phosphatase that can act as a positive and a negative regulator of TCR signal transduction, depending on the nature of the stimulus [62,64]. First, we used an Ab that recognized all the alternatively spliced isoforms of CD45 (Figure 4A first row). Results (Western blots) showed that the expression of CD45 was lower in resting T cells of young subjects compared to elderly individuals when normalized over β-actin (Figure 4B, *p* < 0.01) and remained relatively unchanged over the course of the experiments. The immunoprecipitated CD45 activity was measured and found to be similar in the resting states of T cells of both groups of donors but was significantly higher in T cells of aged donors, 30 s (*p* < 0.05) and 30 min (*p* < 0.01) following activation (Figure 4B, right panel). In young subjects the CD45RA/CD45RO T cell ratio was higher (*p* < 0.01) than in T cell of elderly subjects (Figure 4C, top panel), which is expected based on the higher frequency of memory cells (identified as CD45RO-expressing cells). We also measured the activity of the immunoprecipitated CD45RA and CD45RO. It was found that their activities did not differ between the young and elderly subjects at any time points. (Figure 4C, bottom and middle panels, respectively). Thus, the increased enzymatic activity of total CD45 in elderly donors reflects the increased expression of CD45RO*.* We made a Ponceau to determine that at all measurement points the immunoprecipitated protein amount was the same. Data for these latter experiments were carried out on isolated T cells from 5 independent subjects in each age group in triplicate. Confocal microscopy experiments showed that CD45 location to lipid rafts in T cells of young and elderly donors remained respectively unchanged over the course of the experiments (data not shown).

### Expression, activity and distribution of phosphorylated SHP-1 in T cells with aging

**Figure 5:**
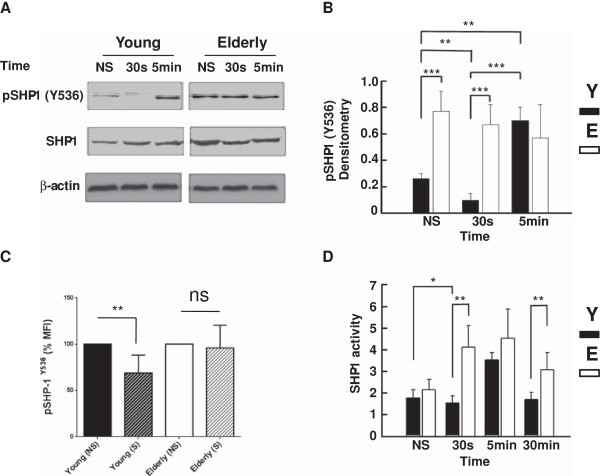
**Western blot analysis of the levels of (active) tyrosine-phosphorylated (Y536) SHP-1. (A)** Purified T cells of young and elderly donors were left non-stimulated (NS) or exposed to a mixture of anti-CD3 and anti-CD28 (5 μg/ml each) mAbs for 30 s or 5 min, as indicated. Cell lysates were sized by SDS-PAGE under reducing conditions, electrotransfered to nitrocellulose membranes and proteins revealed using an anti-Y536 SHP-1 mAb and the chemiluminescence technique. β-Actin was used as control of gel loading. The protein transferred to nitrocellulose membranes were stained with Ponceau to verify that similar amounts of protein had been loaded in each lane. **(B)** Semi-quantitative densitometric analysis of protein bands of young (filled columns) and elderly (empty columns) donors reported in arbitrary units. Data are represented as the mean ± SD and are representative of one of 20 independent experiments. **(C)** Flow cytometry measurements of pSHP-1-Y536 MFI in T cells of young and elderly subjects at non-stimulated (NS) and stimulated for 30 seconds (S) states as decribed in the Materials and Methods section. The non-stimulated is normalized as 100% and significant difference is shown by **, p < 0.01. **(D)** Determination of SHP-1 activity in T cell lysate immunoprecipitate using 4-nitrophenyl phosphate as a substrate. Data show results of T cells of young (filled columns) and elderly (empty columns) donors reported in optical density units. Data are represented as the mean ± SD. Asterisks correspond to statistical significance (Student’s *t*-test) for *p* < 0.05 (*), *p* < 0.01 (**) and *p* < 0.001 (***). Data are representative of one of 20 independent experiments.

SHP-1 is a cytoplasmic tyrosine phosphorylation-regulated protein phosphatase that displays negative regulatory effects on T cell activation. Whereas phosphorylation of Tyr536 increases its activity several folds [65], phosphorylation of S591 decreases its activity [66]. Here, Western blot analysis showed that pSHP-1 (Y536) levels in T cells of young subjects decreased significantly at 30 s following activation through CD3-CD28 stimulation (*p* < 0.01) compared to the resting state, and returns to original levels (Figure 5A and B), suggesting that TCR stimulation transiently but significantly decreases its activity. In marked contrast, the levels of pSHP-1 (Y536) in T cells of elderly donors were elevated in the resting state and remained high 30 s and 5 min after stimulation (Figure 5A and B) indicating that no modulation occurred in T cells after TCR stimulation. These results were confirmed by Flow Cytometry analysis (Figure 5C). The mean fluorescence intensity (MFI) of the phospho-SHP1 was normalized to 100% for the resting T cells from young and elderly and the corresponding MFI for activated cells (30 s) is shown. As in the WB experiments, there is a significant decrease (p < 0.01) in pSHP-1 in T cells from young subjects whereas almost no change was observed in elderly subjects (Figure 5C). Although SHP-1 activity in immunoprecipitates was not significantly different between young and elderly donors in the resting state (Figure 5D), the SHP-1 activity was significantly increased (2.7-fold) in T cells of elderly donors as compared to young subjects, at 30 s following T cell activation (*p* < 0.01) (Figure 5D). It is of note that in line with SHP-1 decreased tyrosine phosphorylation the SHP-1 activity significantly decreased in T cells at 30 sec after stimulation (p < 0.05), while instead increased in T cells of elderly donors. There were no differences at 5 min between the two groups of donors (Figure 5C). Extending the observations to 30 min revealed that SHP-1 activity returned to basal levels in T cell lysates of young subjects but SHP-1 activity was still 1.8-fold higher in the case of elderly subjects (*p* < 0.01) (Figure 5C).

**Figure 6:**
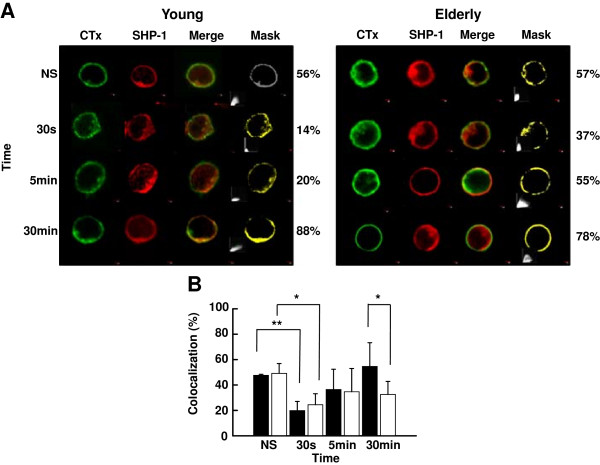
**Confocal analysis of the distribution of SHP-1 in plasma membrane lipid rafts. (A)** Analysis of the distribution in lipid rafts of cholera toxin B subunit (CTx) and SHP-1 of non-stimulated (NS) and activated (anti-TCR/anti-CD28 mAbs, 5 μg/ml each) T cells of young and elderly donors for various periods of time. An illustrative example of individual, merged images and masking is shown. **(B)** Colocalization data showing results of T cells of young (filled columns) and elderly (empty columns) donors. Data are represented as the mean ± SD of pixel intensities determined by masking. The asterisks indicate significance (Student’s *t*-test) for *p* < 0.05 (*) and *p* < 0.01 (**). Data are representative of one of 20 independent experiments.

SHP-1 is partially located in lipid rafts in murine thymocytes and T cell lines [50] and retained in lipid rafts of human neutrophils of elderly donors [67]. Here, SHP-1 was detected to the same extent in lipid rafts of T cells of young and elderly individuals in the resting state (Figure 6A and B). Activation of the cells triggered a significant decrease of SHP-1 localization in lipid rafts at 30 s in the case of both groups of donors, however this was more important in the case of young subjects (p < 0.01) compared to elderly subjects (p < 0.05), followed by recruitment back to lipid rafts (5/30 min) (Figure 6A and B).

### Inhibition of SHP-1 leads to recovery of TCR/CD28-dependent proliferative response and IL-2 production in T cells of aged individuals

**Figure 7:**
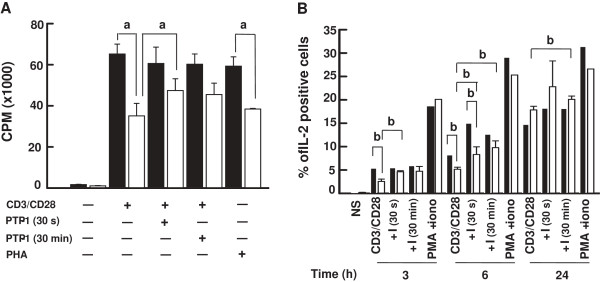
**Proliferation and interleukin-2 production in PBMCs exposed to the SHP-1 inhibitor PTP-1. (A)** PBMCs were isolated from young (filled columns) and elderly (empty columns) donors, left untreated or exposed to the SHP-1 inhibitor PTP-1 (50 ng/ml) for the indicated times. The cells were non-stimulated (NS) or cultured in the presence of a mixture of anti-CD3 and anti-CD28 (5 μg/ml each) mAbs, or PHA (5 μg/ml) for 72 h. Cell proliferation was assayed by incorporation of [^3^H]thymidine. Data are represented as the mean ± SD. Letter (a) corresponds to statistical significance (one way ANOVA) for *p* < 0.05. Non-significance was observed for data relative to results obtained in PBMCs stimulated in the absence of PTP-1. Data are representative of one of 9 independent experiments using different donors. **(B)** Similar conditions of PBMCs treatment in the absence or presence of the inhibitor (I) PTP-1 with the mixture of anti-CD3/anti-CD28 (5 μg/ml each) mAbs or a combination of PMA (50 ng/ml) and ionomycin (iono) (1 ng/ml) were performed for the periods of time indicated. The extent of IL-2 production in non-stimulated (NS) or stimulated T cells was determined by FACS analysis of permeabilized cells using a FITC-labelled anti-Il-2 mAb. Results obtained in the case of young and elderly donors are shown using filled and empty columns, respectively. Letter (b) corresponds to statistical significance (one way ANOVA) for *p* < 0.05. Data are representative of one of 5 independent experiments.

A key feature of altered T cell responses of elderly subjects is a decreased proliferation in response to polyclonal and TCR-directed activation [6,7]. The bulk of the results presented above suggested that the modulation of SHP-1 activity could have a positive influence on T cell proliferation isolated from elderly donors. T cells of both groups of donors were left untreated or treated with a SHP-1 inhibitor (PTP-1) also known to inhibit the PTP1B which is involved in the insulin signaling pathway. We tested proliferation in response to a combination of anti-CD3 and anti-CD28 mAbs. Results showed that the proliferation of T cells of elderly donors in the absence of PTP-1 was significantly decreased (53.8%) in response to stimulation through the CD3-CD28 pathways as compared to young donors (Figure 7A). Lymphocyte response to polyclonal PHA activation was also decreased (64.8%) in the case of aged donors, as reported [5]. However, treatment of the cells with PTP-1 (50 ng/ml) for 30 s resulted in a partial but significant restoration (1.35-fold) of T cell proliferation of elderly donors, which raised the proliferative response to levels not significantly different from that of young donors (one-way ANOVA, *p* > 0.27). The proliferative T cell responses of elderly donors did not increase further when the cells had been exposed to PTP-1 for 30 min and was not different than that observed in the case of T cells of young donors treated with PTP-1 for 30 s (Figure 7A).

We next sought to determine whether the recovery of T cell proliferation of aged donors correlated with increased intracellular production of IL-2. Results from flow cytometry experiments showed an increase in the percentage of IL-2-producing T cells of young and elderly subjects measured 3 h, 6 h and 24 h after CD3-CD28 stimulation (Figure 7B). The percentage of IL-2-positive stimulated T cells of young donors was significantly higher than in the case of aged donors at 3 h and 6 h but was similar after 24 h of stimulation (Figure 7B). A brief (30 s) treatment with PTP-1 did not induce T cells of elderly donors to produce IL-2 to the same extent as T cells of young donors at 6 h only (p < 005). However, the presence of the SHP-1 inhibitor resulted in significant increases in the percentage of IL-2-positive T cells with respect to the absence of inhibitor in stimulated T cells of aged donors at 3 h, 6 h and 24 h following activation (Figure 7B). A similar effect was observed in T cells of young donors except at 3 h. Extended exposures to PTP-1 (30 min) resulted in IL-2 production that was not significantly different between the two groups of donors when measured 3 h, 6 h and 24 h following T cell stimulation (Figure 7B).

### Effect of SHP-1 inhibition on Lck phosphorylation in T lymphocytes of young and elderly individuals

**Figure 8:**
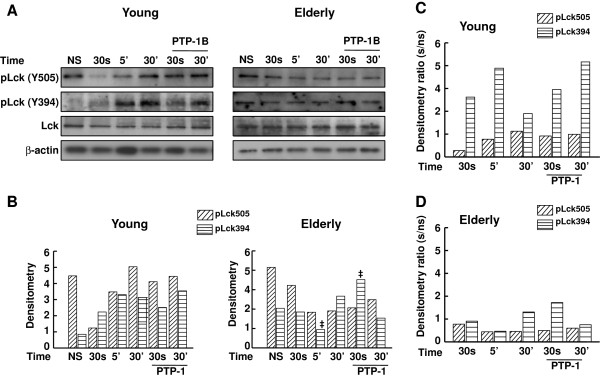
**Western blot analysis of the levels of Y505- and Y394-phosphorylated forms of Lck in untreated and PTP-1-treated T cells. (A)** T cells were left non-stimulated (NS) or exposed to a mixture of anti-CD3 and anti-CD28 (5 μg/ml each) mAbs for the times indicated, in the absence of the SHP-1 inhibitor (I) PTP-1 or its presence (50 ng/ml). Cell lysates were sized by SDS-PAGE under reducing conditions, electrotransfered to nitrocellulose membranes and proteins revealed using relevant mAbs and the chemiluminescence technique. β-Actin was used as control of gel loading. The protein transferred to nitrocellulose membranes were stained with Ponceau to verify that similar amounts of protein had been loaded in each lane. **(B)** Densitometric semi-quantification of the data, shown in arbitrary units in the case of young and elderly donors. For sake of clarity, significant statistical differences are indicated with filled circles (●, pLck-Y505) or filled squares (■, pLck-Y394) with respect to resting cells in the case of young donors. In the case of elderly donors, significant statistical differences are indicated with filled diamonds (♦, pLck-Y505) or ddags (‡, pLck-Y394) with respect to resting cells. **(C and D)** Densitometric ratio with respect to resting cells were determined for the various times and conditions of experiments for young **(C)** and elderly **(D)**. Data are representative of one of 15 independent experiments.

We assessed the time-dependent levels of active (Y394) and inactive (Y505) forms of pLck in response to stimulation through CD3-CD28 in T cells of young and elderly donors exposed or not to PTP-1. Western blot analysis showed that the elevated levels of Lck-Y505 in resting cells of young donors decreased rapidly 30 s after activation (*p* < 0.05), then steadily increased 5 and 30 min later (Figure 8A and B). As expected, the levels of pLck-Y394 were low in resting cells of young donors, rapidly increased 30 s and 5 min after activation and remained significantly sustained for the next 30 min (p < 0.01) (Figure 8A and B). Treatment with the PTP-1 did not appear to further increase the activated form (Y394) of Lck when assessed 30 s and 30 min after T cell activation, suggesting that the optimal activation of early signaling through CD3-CD28 stimulation could not be further modulated. In this context, it is to be noted that the inactive form (Y505) of pLck was less sensitive to PTP-1 inhibitory effect likely because pLck-Y394 is the target of SHP-1 [48]. In contrast, the pattern of pLck dynamic in activated T cells of aged donors was vastly different. Whereas the levels of inactive pLck-Y505 were elevated in resting cells, they very slowly but significantly decreased (p < 0.05) over time after activation (Figure 8A and B) to remain steady 5 and 30 min after activating the cells. The levels of active pLck (Y394) did not change 30 s after stimulating the cells as compared to resting cells (Figure 8A and B). There was a decrease at 5 min, followed by an increase at 30 min. The presence of PTP-1 significantly increased the levels of activated pLck (Y394) very early (30 s) following T cell activation (p < 0.01) (Figure 8A and B) showing that inhibition of SHP-1 can modulate the positive activation of Lck in aged donors. Differences between lymphocyte responses to activation were further revealed by doing a ratio between the two phosphorylated forms of Lck in activated T cells with respect to resting cells. This approach showed that the ratio of pLck-Y505 was low in resting cells of young donors and remained relatively low over the time of experiments, even after prolonged (30 min) activation (Figure 8C). As expected, T cell activation resulted in a rapid increase of the ratio of pLck-Y394 after 30 s which was further increased after 5 min but which decreased after prolonged (30 min) activation (Figure 8C). Treatment with PTP-1 maintained the active form of Lck over time (Figure 8C). A significantly different pattern of pLck behavior was observed in the case of aged donors (Figure 8D). In this case, the levels of inactive and active Lck were low under all experimental conditions, suggesting a sluggish and altered response in T cells activated through CD3-CD28 in aged donors. However, the presence of PTP-1 favored Lck activation only after 30 s of stimulus in the case of elderly subjects (Figure 8D).

## Discussion

Here, we have tested the hypothesis that the T cell pool in elderly subjects displayed reduced functional capacities due to altered negative feedback mechanisms that are involved in the regulation of the early steps of T cell activation. Data presented here are based on purified T cells and total T cell populations of young and elderly individuals. Immune responses become less efficient as humans get older and this phenomenon contributes to the overall state of immunosenescence. In this connection, accumulated data [36,40,41,68] have established that Lck is a pivotal regulator of the TCR- and CD28-associated early events of T cell activation and subsequent signal transduction, gene expression and T cell proliferation [28]. Data presented here provide evidence that SHP-1 exerts a greater negative feedback effect on Lck-mediated activation of T lymphocytes of aged humans than in T cells of young individuals. This accrued effect of Lck in aging may, at least in part, be responsible for the characteristic impaired proliferation of these cells in response to stimulation.

The levels of the inactive phosphorylated form (Y505) of Lck relative to total Lck were moderate in the resting state of T cells of young donors, as reported in T cells and the Jurkat T cell line [69]. The levels of pLck (Y505) decreased over time of stimulation, suggesting that the levels of inactive kinase decreased presumably at the expense of the active form. In marked contrast, the levels of Lck Y505 only slightly decreased over the time course of stimulation in the case of elderly individuals, suggesting a lack of modulation or maintenance of the inhibitory status of Lck. This differential behavior of T cell response in young and elderly donors suggested an alteration of T cell regulation in the control loop of Lck activation involving the PAG-Csk complex [35,46,61] in elderly subjects. An alternative but not exclusive interpretation was an alteration in the efficiency of CD45 to remove the phosphate group at position Y505 of Lck. Engagement of TCR triggers dephosphorylation of PAG and the subsequent migration of Csk out of the lipid rafts, a situation which facilitates CD45-dependent removal of the phosphate group on Y505 [70] and pLck-Y394 upregulation. We showed here that several aspects of the PAG/Csk regulation loop were altered in T cells of elderly subjects. First, Western blot analysis clearly showed that the levels of pPAG were elevated in resting T lymphocytes of elderly subjects and remained at higher levels than those observed in young donors over the course of the experiments (Figure 2). These observations suggested that in T cells of aged subjects, the elevated levels of pPAG kept Lck in its inactive phosphorylated form, in agreement with the results of Western blots (Figures 1A). Second, this interpretation was further supported by analysis of location of pPAG in lipid rafts which clearly showed a differential distribution in T cells of elderly and young individuals (Figure 3). Kinetically, pPAG migrated out of lipid rafts in response to stimulation in the case of T cells of young donors but the levels of pPAG in lipid rafts remained unchanged in resting and activated T cells of aged donors (Figure 3). The inability of pPAG to migrate out of lipid raft microdomains in the case of T cells of aged donors was likely related to the reported decreased function of lipid rafts of T cells of elderly individuals due to the increased cholesterol level [54]. Our data fit current models [28,68,71] whereby TCR/CD28 activation induces cellular phosphatases to dephosphorylate Csk-associated pPAG (Y317) during concurrent trafficking out of lipid rafts, resulting in the release of Csk to the cytoplasm. Interruption of Csk-pPAG association results in abrupt decline of phosphorylated (Y505) Lck. Our data showed differential levels of pLck (Y505) in young and elderly subjects that explained the decreased response in T cells of aged individuals [55].

CD45 activity controls the upregulation of Lck and Fyn activation [71-73]. Here, an alteration in CD45 activity did not appear to be the major cause of the differential activation of Lck in the two groups of individuals. Unexpectedly, the levels of CD45 or its activity were higher in activated T cells of elderly subjects (Figure 4) but there was no differences in lipid raft location of both groups of donors. Also, unexpectedly CD45RA expression and activity did not depend on the age of the subjects, while the expression of CD45RO (marker of memory T cells) was higher in T cells of elderly. This may contribute to the increase of the total CD45 expression and activity at least at 30 s of stimulation (Figure 4A through C). As there is no clear dichotomy in the role of CD45 isoforms in reduced T cell activation with aging, we can hypothesize that the isoform itself has a minimal importance while the associated signalosome, starting with Lck activation is more important. We have previously shown that the formation and composition of membrane lipid rafts, an important step of cell activation, are altered in T cells from elderly individuals.

SHP-1 and CD45 play gatekeeper functions as fine regulators of T cell activation [62,64,73]. Whereas CD45 is generally considered as having a positive and essential role in T cell activation, SHP-1 acts as a negative feedback mechanism by targeting tyrosine-phosphorylated components associated with the early events of T cell signaling such as LAT [74], Lck [48], ZAP-70 and the ξ homodimer [75]. SHP-1-dependent dephosphorylation of these substrates leads to inhibition of T cell activation. Here, the levels of the active form (Y536) of SHP-1 significantly decreased 30 s after TCR-CD28 stimulation of T cells of young donors (Figure 5), in keeping with positive Lck upregulation and T cell activation. However, the active form of SHP-1 returned to elevated levels after 5 min, in agreement with the fact that T cells become rapidly committed to activation following engagement of the TCR and CD28. In marked contrast, the levels of SHP-1-Y536 remained elevated in T cells of elderly individuals, suggesting that SHP-1 played a negative role in the early sequence of events leading to activation of T cells in aged donors. This observation suggested that SHP-1 remained active under TCR/CD28 stimulation leading to maintenance of Lck in an inhibitory state. The interpretation of a persistent dominant negative role of SHP-1 in T cells of aged donors was further supported by determination of its activity. SHP-1 activity was similar in resting T cells of both groups of donors but rapidly increased and remained high in stimulated T lymphocytes of elderly individuals. In contrast, SHP-1 activity first significantly decreased and became elevated later (5 min) after activation in lymphocytes of young individuals (Figure 5). SHP-1 is mostly located outside of lipid rafts in Jurkat T cells but recruitment increases in response to protein tyrosine phosphatase inhibition [76]. Furthermore, targeted recruitment of SHP-1 in lipid rafts results in dephosphorylation of LAT and subsequent defects in downstream events of TCR signaling in Jurkat T cells [74]. Here, results of confocal analysis revealed that SHP-1 was present to the same levels in resting T cells of young and elderly donors, migrated out of lipid rafts more importantly in T cells from young subjects than in T cells of elderly after activation, but returned to lipid rafts over time (Figure 6). Thus, partitioning dynamics of SHP-1 in lipid rafts may to some extent also contribute to the decreased proliferative response of T cells of aged individuals, concomitantly with the much pronounced age-related differences of SHP-1 activity.

The bulk of the data pointed toward SHP-1 as a key protein phosphatase that negatively modulated T cell proliferation in aged individuals. We tested whether inhibition of SHP-1 would improve the proliferative response of lymphocytes of elderly individuals to CD3-CD28 stimulation. As previously reported, T cells of elderly donors displayed an impaired proliferative response to TCR-CD28-dependent activation and to mitogenic stimulation (Figure 7A). In marked contrast, treating the cells with an SHP-1 inhibitor (PTP-1) for 30 s or 30 min upregulated T cell proliferation to levels that were not statistically different (one-way ANOVA) than those found in cells of young donors. The observation that the presence of PTP-1 did not further increase CD3-CD28-dependent T cell response of young subjects can be explained by the absence of an effect on pLck-Y394 levels thus, on the already maximal proliferation of these cells. IL-2 production was increased in lymphocytes of aged donors in experiments using PTP-1 (Figure 7B). These observations suggested that SHP-1 was a key negative regulator of the proliferative response of T cells associated with aging (Figures 7 and 8) and that its inhibition could efficiently restore two of the most altered functions observed with aging in T cells.

Age-related changes in T cell subset composition involve a reduced frequency/number of naïve T cells, increased number of memory T cells and increased proportion of CD28^-^ T cells, especially in the CD8^+^ subpopulation [13]. A decrease in CD28 expression would affect the efficiency of the co-stimulatory pathway and this situation has been suggested to be in part responsible for impaired T cell activation with aging [77]. On the other hand, data from our laboratories have provided evidence for a direct correlation between the state of activation of Lck and LAT and their association/recruitment with lipid rafts of CD4^+^ and CD8^+^ T cells [54]. In relation with these reports, recent data have provided evidence that, in addition to being a required component of TCR/CD3 signaling, Lck is an obligatory link for T cell activation through the CD28 co-stimulatory pathway. Kong *et al.*[41] have put forward a model whereby activated Lck associates with pY207 of the C-terminal portion of CD28 through its SH2 domain. This association retains Lck in lipid rafts and allows its concomitant interaction with GLK-dependent phosphorylated PKCθ through its SH3 domain. The model predicts that Lck therefore provides an essential molecular bridge between CD28 and PKCθ. Our data suggest that interfering with the negative regulatory effect of SHP-1 would have a beneficial effect on signals 1 and 2 of T cell activation and would help to restore T cell proliferation in aging, as shown here (Figure 7). Furthermore, it has been recently reported that impairment of early signalling events in activated T cells allows prediction of the levels of expression of the co-stimulatory molecules CD28 and CD27. This situation further predicts the number of population divisions in culture from a limited subset of signalling molecules such as Lck [78].

## Conclusion

**Figure 9:**
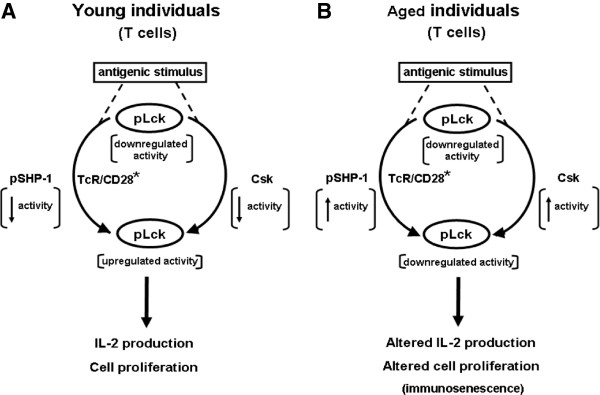
**Minimal model summarizing the roles of SHP-1 and PAG in negative regulation of T cell response in aging. A)** The model proposes that in T cells of young individuals, ligation of the TCR and CD28 leads to activation (TCR/CD28^*^) and concomitant up-regulation of Lck activity resulting from dephosphorylation of Y505, subsequent release of self-inhibition and activation following autophosphorylation at position Y394. Activation of pLck leads to phosphorylation of ITAM motifs of CD3 and the ζ homodimer, triggering the early events of signal transduction. Data reported here showed that the activity of pSHP-1 in T cells of young subjects was transiently low following T cell activation, whereas pPAG transiently migrated out of lipid rafts. These combined events would favor maintaining upregulation of Lck activity which would fulfill the requirements of Signal 1 of T cell activation. Initiation of the Signal 2 leads to full T cell response. **B)** A similar series of events occurs in T cells of elderly individuals. However, data reported here showed that the activity of pSHP-1 was sustained in these cells and that pPAG was retained in lipid rafts thus contributing to sustained elevated Csk activity. These combined events would lead to lowered activity of pLck, a decrease of the strength of Signal 1 and, an overall decrease of T cell activation. These conditions lead to a diminished T cell response that contributes to immunosenescence.

Overall, our observations are in agreement with previous reports that SHP-1 acts as negative regulator of T cell signaling [52,53,74,75,77,79]. In addition, a recent report has linked Csk to the termination or control of TCR signaling through modulation of Lck activity [46]. The authors showed that feedback circuits involving Csk, CD45 and, possibly, the inhibitory adaptor protein Dok-1 were key regulators of the early events of T cell activation. These circuits could tip the balance in favor of TCR signaling or an altered response depending on the aging process. Furthermore, SHP-1 has been reported to be involved in regulation of other cells of the immune system. For instance, studies of neutrophils have revealed an alteration of GM-CSF modulation of SHP-1 with aging and a defective protection against apoptosis in these cells which were reversed by inhibition of SHP-1 [67]. In addition, it has been recently reported that SHP-1 is a regulator of dendritic cell functions in mice [80,81]. Hyperphosphorylated (active) SHP-1 has recently been reported to regulate BCR signaling in germinal centers to favor selection of higher affinity B cells [82], whereas loss of function in B cells causes systemic autoimmunity [83]. The bulk of the data presented here are in agreement with the interpretation that the Csk/PAG loop and SHP-1 are key negative participants of T cell functions in aging since this negative influence cannot be relieved by TCR/CD28 stimulation (Figure 9). The successful improvement of T cell functions in T cells of elderly donors by inhibiting the SHP-1 activity can be a powerful immunomodulatory tool and requires further investigations. One limitation of the present study is that it cannot discern between internal T cell changes and changes due to the relative abundance of T cell subsets with aging. In particular, it is important to point out that the present study cannot determine if the effect observed is specific for a particular subset of T cells [84] even if our preliminary data suggest that the CD45RA or CD45RO T cell subpopulations cannot explain these changes. Nevertheless, the data provide support for a key role of Lck in the altered response of T cells in aging, revealing additional clues to unravel the underlying mechanisms of immunosenescence which are under current investigations in our laboratories, especially addressing this question in T cell subpopulations.

## Materials and methods

### Antibodies and reagents

Polyclonal antibodies (pAbs) anti-PAG (sc-25748), -Lck (sc-28882), -Csk (sc-286), -SHP-1 (sc-287), -CD45 (sc-1178), -CD45RA (sc-19664), -CD45RO (sc-70712) and -β-actin (sc-1616) were from Santa Cruz Biotechnology (Santa Cruz, CA). Anti-CD3 (clone UCHT1) and anti-CD28 (clone CD28.2) monoclonal antibodies (mAbs) were from BD Biosciences (Mississauga, ON). pAbs anti-phospho-PAG (Y763) (ab18030) and anti-PAG (ab56521) were from Abcam (Cambridge, MA). An anti-phospho-Tyr505 (2751S) Lck Ab was from Cell Signaling Technology Inc. (Pickering, ON) whereas an anti-phospho-Tyr394 (SAB4300118) Lck Ab was from Sigma-Aldrich. The anti-phospho-Tyr536 SHP-1 mAb was from ECM Biosciences (Versailles, KY). A FITC-conjugated anti-IL-2 mAb (clone M1Q_17H12) was purchased from BD Biosciences. The SHP-1 PTP inhibitor I (PTP-I, product #540200) was purchased from Calbiochem (EMD Chemicals Inc., Gibbstown, NJ). Alexa Fluor 488-labelled cholera toxin B subunit and Alexa Fluor 568-labelled goat anti-rabbit IgG were purchased from Invitrogen (Carlsbad, CA). RPMI-1640 culture medium was obtained from Wisent Inc. (St Bruno, QC), whereas Ficoll Paque plus and Dextran T-500 were from GE Healthcare (Piscataway, NJ). Protein A/G-bound Sepharose was obtained from BioVision Inc. (Milpitas, CA). A Western lightning Plus ECL kit and PVDF membranes were purchased from PerkinElmer (Waltham, MA). Reagents for SDS-PAGE were from Bio-Rad (Richmond, CA) and Fisher Scientific (Montreal, QC).

### Subjects

Twenty five healthy elderly volunteers aged 65 to 78 years (mean, 73 years) participated in the study. The cohort of 25 young healthy subjects was 19 to 25 year old (mean, 22 years). The research protocol was approved by the local institutional ethics committee of the Research Center on Aging. All subjects gave written informed consent. The volunteers were in good health, normolipemic and satisfied the inclusion criteria of the SENIEUR protocol for immune investigations of human elderly subjects [85]. Individual experiments were performed in parallel (on the same day), that is by collecting blood samples from the same number of healthy young donors and healthy aged donors.

### Isolation of PBMCs

Blood obtained by venipuncture was collected in heparinized tubes and diluted two-fold with phosphate-buffered saline (PBS). PBMCs were isolated by Ficoll Paque plus density sedimentation, as described [56]. The buffy coat was recovered, the cells were washed (PBS) and counted. Cell viability was greater than 95% (Trypan blue exclusion). Identical numbers of cells from young and aged donors were used in each comparative experiment.

### T cells purification

PBMCs were freed of monocyte by adhesion to plastic tissue culture flasks coated with autologous serum (1 h, 37°C) and, B cells and phagocytic cells by nylon wool retention, as described [86]. Purified T cells were greater than 98% CD3^+^ cells with less than 1% surface IgM (B cells)-, CD16 (NK cells)- and CD14 (monocytes)-positive contaminating cells as verified by FACScan (FACSCalibur). Cell viability was greater than 97% (Trypan blue exclusion). Identical numbers of T cells from young and aged donors were used in each comparative experiment.

### Lymphocyte proliferation

PBMCs (2 × 10^5^ cells/well) were exposed to anti-CD3 (5 μg/ml) and/or anti-CD28 (5 μg/ml) for 72 h in 96 well flat-bottomed microcultures (Microtest, Becton Dickinson) in a final volume of 200 μl of RPMI 1640 medium containing 10% foetal bovine serum (FBS), streptomycin (100 μg/ml) and penicillin G (100 U/ml) at 37°C in an atmosphere of 95% air, 5% CO_2_ and 90% relative humidity. Cell proliferation was quantitated by measuring [^3^H]thymidine incorporation, as described [55].

### Isolation of lipid rafts

T cells were kept for 1 h in RPMI medium at 37°C. The cells (20 × 10^6^ lymphocytes) were then exposed to a combination of anti-CD3 (5 μg/ml) and anti-CD28 (5 μg/ml) mAb for various periods of time at 37°C, as described [63] or were left untreated (control). Lipid raft isolation on sucrose density gradients (9 fractions) was done as already described [87]. Lipid rafts were distributed in fractions 1 to 3 of the gradient whereas non-lipid rafts corresponded to fractions 7 to 9. Western blotting analyses were done using pooled lipid raft and non-lipid raft fractions.

### Western blots

Proteins from total cell lysates (20 μg) or pooled lipid raft fractions (30 μl) were sized by SDS-PAGE under reducing conditions, transferred to PVDF membranes and revealed by Western blotting, as described [63,87]. Acrylamide concentration was 10% in SDS-PAGE analysis performed under reducing conditions, according to Laemmli [88]. To reveal β-actin, PVDF membranes were stripped of antibody by washing (twice, 5 min) with TBST buffer (20 mM TRIS, 150 mM NaCl, 0.1% Tween 20, pH 7.4), incubation with PBS (pH 2.0, ajusted with HCl) for 40 min at 65°C, followed by washing (twice, 5 min) with TBST. The membrane was then probed with an anti-actin antibody. Densitometric analyses were performed using the image analyzer Chemigenius2 Bio Imaging System (Syngene, Frederick, MD) or the Java-based ImageJ freeware (http://rsbweb.nih.gov/ij/). All blots were first normalized towards actin and then to the non-phosphorylated form of the studied protein.

### Confocal microscopy analysis

T cells in RPMI 1640 medium containing 10% FBS were exposed to a combination of anti-CD3 (5 μg/ml) and anti-CD28 (5 μg/ml) mAbs for various periods of time at 37°C. Stimulation was terminated by centrifugation, the cells were washed with culture medium and then incubated for 30 min (4°C) with Alexa Fluor 488-conjugated cholera toxin B subunit. They were then fixed with 4% paraformaldehyde in PBS for 15 min on ice. After a wash with cold PBS, the cells were incubated for 1 h at room temperature in the presence of the relevant primary antibodies in PBS containing 1% skimmed powdered milk, 1.4% bovine serum albumin (BSA) and 0.1% Triton X-100. T cells were then washed with PBS and incubated in the same solution containing the secondary Ab (Alexa Fluor 568-labelled goat anti-rabbit IgG) for 1 h at room temperature. After washing with ice-cold PBS, the cells were placed on microscope slides and mounted with Vectashield Mounting Medium (Vector Laboratories, Burlington, ON). Scanning confocal microscope analysis were done as published using a FV1000 instrument (Olympus, Tokyo, Japan) coupled to an inverted Olympus microscope with a 63X oil immersion objective [87]. Specimens were laser-excited at 488 nm (40 mW argon laser) and 563 nm (helium-neon laser). Serial horizontal optical sections of 512 x 512 pixels were taken at 0.5 μm intervals through the entire thickness of the cell. Images were acquired typically from 10–15 cells of similar size from each experimental condition using identical settings of the instrument. In the case of Alexa 488/Alexa 563-merged fluorescence images, dot fluorograms were obtained by plotting pixel values of each fluorochrome toward the horizontal and vertical axis, respectively. Quadrant markers were used to separate staining in background pixels (C, lower left), red-only pixels (A, upper left), green-only pixels (D, lower right) and co-localizing pixels (B, upper right). Percentage of colocalization was assessed as follows: (number of pixels in quadrant B)/(number of pixels in quadrant B + number of pixels in quadrant D). Images were contrast-enhanced, pseudocolored according to their original fluorochromes, merged (co-localizing green and red pixels are in yellow pseucolor in the Figures), cropped and then assembled using the FluoView version 3.1 software (Olympus, Tokyo).

### Protein tyrosine phosphatase assays

T cells (5 x 10^6^ cells/ml) in RPMI 1640 medium were left untreated or treated with PTP-1 for different periods of time, as follows. In the cases of short time experiments (30 s and 5 min), the cells were distributed in Eppendorf tubes (2 ml of cell suspension/tube) and PTP-1 was added to a final concentration of 50 ng/ml. For each set of experiments, T cells were centrifuged, washed and suspended in RPMI 1640 medium (10 x 10^6^ cells/ml) and exposed to a mixture of anti-CD3 (5 μg/ml) and anti-CD28 (5 μg/ml) mAbs for the times indicated in the relevant Figures. In the cases of long time experiments (30 min), PTP-1 treatments were done with cells resting in 6-well culture plates (10 x 10^6^ cells/well) containing 2 ml of culture medium in each well. At the end of the treatment, lymphocytes were retrieved, centrifuged and washed. Anti-CD3-CD28 stimulation was done as described above. The cells were then washed with PBS, and resuspended in ice-cold lysis buffer (150 mM NaCl, 10 mM EGTA, 5 mM EDTA, 1 mM phenylmethylsulfonyl fluoride (PMSF), 10 mM sodium polyphosphate, 1% Nonidet P-40 (NP-40) and antiproteases cocktail in 50 mM (4-(2-hydroxyethyl)-1-piperazineethanesulfonic acid (HEPES), pH 7.4) for 30 min at 4°C, with periodic gentle shaking. Lysates were cleared by centrifugation (13,000 rpm). Samples containing 120 μg of proteins were retrieved and volume completed to 350 µl Protein A/G-bound Sepharose (20 μl) beads were added for preclearing. The mixture was gently rocked for 30 min at 4°C mAbs directed against SHP-1 or CD45 or CD45RA or CD45RO (2 μg/experimental condition) were added to the supernatants, followed by incubation overnight at 4°C under gentle rocking (2 μg/experimental condition). Protein A/G-bound Sepharose (35 μl) beads were added, and incubations under gentle rocking were performed for 4 h at 4°C. The beads were washed with buffer (150 mM NaCl, 10 mM EGTA, 5 mM EDTA and 0.1% NP-40 in 5 mM Tris.HCl buffer, pH 7.5) and subjected to phosphatase assays. Briefly, the beads were washed once with assay buffer (0.5 mM EGTA in 25 mM HEPES, pH 7.0) and then incubated with 200 μl of the same buffer containing 10 mM 4-nitrophenyl phosphate at 37°C for 4 h with periodic mixing. Reactions were stopped by addition of 0.2 M NaOH (800 μl), beads were removed by brief centrifugation, supernatants were distributed in 96-well plates and absorbance was read at 405 nm using a Victor X5 2030 Multilabeled reader (PerkinElmer (Waltham, MA). Equal amounts of proteins (Bradford’s reagent, Bio-Rad, Richmond, CA) from young and elderly donor aliquots were used in CD45, CD45RA, CD45RO and SHP-1 phosphatase assays, as already described [67] as verified also by Ponceau staining.

### Measurement of intracellular IL-2

PBMCs (1 x 10^6^ cells/ml) in RPMI 1640 medium were left untreated or treated with PTP-1 for different periods of time, as described for protein phosphatase assays. They were stimulated using a combination of anti-CD3 (5 μg/ml) and anti-CD28 (5 μg/ml) mAbs for the times indicated in the legend of the Figure. In the case of each experiments, the cells were placed in Eppendorf tubes, washed by brief centrifugation with cold PBS, fixed by treatment (20 min in the dark, 4°C) with 250 μl of 4% (w/v) paraformaldehyde (BioLegend, Burlington, ON) and then washed with a mixture of PBS (1 ml) and diluted (PBS) permeabilization buffer (250 μl) containing FBS and saponin (PermWash buffer, BD Pharmingen). After washings, staining was done with permeabilization wash buffer (200 μl) containing a FITC-conjugated rat anti-human IL-2 Ab (0.2 μg/ml) for 30 min at 4°C, in the dark as already described [56]. The stained cells were washed once with permeabilization wash buffer and resuspended in PBS (250 μl). The cells were analyzed within 24 h by flow cytometry using a FACSCalibur instrument (Beckton Dickinson). A minimum of 10,000 events were acquired in each analysis.

### Flow Cytometry measurement of pLck-Y505 and pSHP1-Y536 in T cells after TCR/CD28 stimulation

After stimulation for 0, 30 s and 5 minutes, cells (1 x10^6^) were suspended into 500 μL of PBS 1X at room temperature (RT), and fixed 10 minutes in the dark at room temperature (RT) with 1% paraformaldehyde pre-warmed (Biolegend, Burlington, ON). Then cells were saturated by an incubation of 10 minutes with 10% PBS-AB-human serum (Life technologies). After two washings, a 30 minutes surface staining was made with anti-CD3 Alexa700, CD45RA BV421, and CD45R0 FITC (BD Biosciences, Mississauga, ON). After two washings and vortex of the sediment, 1 mL of BD Phosflow Perm Buffer III was added and incubated for 30 minutes at 4°C. Three washing with 3 mL of PBS was performed before starting 1 hour of intracellular staining at RT in the dark. Antibodies used were: pLckY505 Alexa Fluor 647 (BD Biosciences, Mississauga, ON) and pSHP1 (ECM Biosciences, Versailles, KY) with anti-rabbit IgG PE (Ebioscience, San Diego, CA) were used. The second intracellular staining with anti-rabbit IgG PE was made 1 hour at RT in the dark. After washing, cells were ready to be analysed on a FACS Aria III cytometer (BD Biosciences).

### Statistical analyses

Data were analyzed using Student’s *t*-test and one-way ANOVA (SigmaStat software, Systat Software Inc., Chicago, IL).

## Competing interests

The authors declare that they do not have any competing interests.

## Authors’ contributions

ALP, CF, HG and CTTY carried out the bulk of the experiments. NA and KT contributed to the studies by performing proliferation experiments and some of the Western blots. AL, GD and TF collaborated in the design, analysis and critical review of the data with the other authors. GD and TF wrote most part of the manuscript. All authors read and approved the final manuscript.
